# Treatment of subcutaneous abdominal wound healing impairment after surgery without fascial dehiscence by vacuum assisted closure™ (SAWHI-V.A.C.®-study) versus standard conventional wound therapy: study protocol for a randomized controlled trial

**DOI:** 10.1186/1745-6215-14-394

**Published:** 2013-11-20

**Authors:** Dörthe Seidel, Rolf Lefering, Edmund AM Neugebauer

**Affiliations:** 1 IFOM - Institute for Research in Operative Medicine, Faculty of Health - School of Medicine, University of Witten/Herdecke, Ostmerheimer Strasse 200, Building 38, 51109, Cologne, Germany

**Keywords:** Surgical wound, Wound healing, Wound healing disorder, Wound healing impairment, Post-surgical wound healing disorder, Surgical wound infection, Abdominal wound healing impairment, Vacuum assisted closure, Negative pressure wound therapy, Abdominal wall

## Abstract

**Background:**

A decision of the Federal Joint Committee Germany in 2008 stated that negative pressure wound therapy is not accepted as a standard therapy for full reimbursement by the health insurance companies in Germany. This decision is based on the final report of the Institute for Quality and Efficiency in Health Care in 2006, which demonstrated through systematic reviews and meta-analysis of previous study projects, that an insufficient state of evidence regarding the use of negative pressure wound therapy for the treatment of acute and chronic wounds exists. Further studies were therefore indicated.

**Methods/design:**

The study is designed as a multinational, multicenter, prospective randomized controlled, adaptive design, clinical superiority trial, with blinded photographic analysis of the primary endpoint. Efficacy and effectiveness of negative pressure wound therapy for wounds in both medical sectors (in- and outpatient care) will be evaluated. The trial compares the treatment outcome of the application of a technical medical device which is based on the principle of negative pressure wound therapy (intervention group) and standard conventional wound therapy (control group) in the treatment of subcutaneous abdominal wounds after surgery. The aim of the SAWHI-VAC® study is to compare the clinical, safety and economic results of both treatment arms.

**Discussion:**

The study project is designed and conducted with the aim of providing solid evidence regarding the efficacy of negative pressure wound therapy. Study results will be provided until the end of 2014 to contribute to the final decision of the Federal Joint Committee Germany regarding the general admission of negative pressure wound therapy as a standard of performance within both medical sectors.

**Trial registration:**

Clinical Trials.gov NCT01528033

German Clinical Trials Register DRKS00000648

## Background

### The medical problem

Wound healing impairment after surgical procedures is a common problem in health care. Complications associated with surgical incisions range from the annoying to the life threatening, and can be more common and serious than those related to the primary surgical procedure. These complications include wound healing disorders caused by factors due to the surgical procedure itself, by patient-related factors, or both. These factors are typically interrelated, complex in nature, and often difficult to prevent or to treat successfully. The most common post-operative wound healing impairments are seroma or hematoma, necrosis of wound margins or adjacent soft tissue, infections, all with potential to cause dehiscence of the primarily closed wound. The occurrence of wound healing impairment of the abdominal wall represents a multidisciplinary, treatment- and cost-intensive problem in clinical life whether it is caused by infectious or non-infectious factors [1-3]. Only a few clinical trials have been conducted regarding the use of negative pressure wound therapy (NPWT) for subcutaneous abdominal wound healing impairment [4-7] or for example, the adjunctive management of abdominal wounds with exposed and known infected synthetic mesh [8]; thus, a randomized controlled clinical trial (RCT) of high quality that is able to make a statement concerning efficacy and effectiveness of this innovative therapy option is still lacking.

### Vacuum-assisted wound closure (V.A.C.®)

Basic science studies demonstrated positive effects of NPWT on wound healing [9,10] and many case reports and case series document broad use of V.A.C.® (http://www.kci-medical.co.uk/uk-eng/home) in various clinical settings [11].

Regarding the clinical results and mechanisms of action of NPWT, the literature provides evidence with regards to V.A.C.® Therapy’s effectiveness in chronic wounds such as in diabetic foot wounds, pressure ulcers and lower extremity ulcers [12]. Additionally, V.A.C.® Therapy is used in the treatment of a variety of acute wounds, including abdominal wounds [13], surgical and dehisced wounds [13-15], grafts and flaps [16,17], deep sternal wounds [18-20] and partial thickness burns [21,22].

### Recent history

When compared to standard conventional wound therapy (SCWT), most of the published literature confirms the advantage of negative pressure wound therapy (NPWT), also called vacuum assisted wound closure, but sufficient proof of efficacy and effectiveness is lacking [12,23]. The Federal Joint Committee (G-BA) is the highest decision-making body of the joint self-government of physicians, dentists, hospitals and health insurance funds in Germany. The G-BA faced this situation by commissioning a report on efficacy and effectiveness of NPWT by the Institute for Quality and Efficiency in Health Care (IQWiG). The final IQWiG-reports [24,25] concluded that, in spite of some indications that NPWT may improve wound healing, the methodological quality of the so far performed trials is unsatisfactory and thus the body of evidence available is insufficient, more specifically in the post-acute care setting. In 2011, Peinemann and Sauerland updated the systematic literature review [26] and concluded that although there may be a positive effect, clear evidence for efficacy and effectiveness of NPWT is still missing and RCTs of high quality are still needed to evaluate the benefit of this wound treatment option. Since the updated review, only a few RCTs on NPWT with the objective to achieve complete wound closure have been performed [27,28]. Thus, there is still a need for a RCT of high quality to contribute to the decision about the benefit of the use of NPWT for wound healing.

### Study objectives and purpose

This RCT was designed to evaluate the efficacy and effectiveness of V.A.C.® Therapy for the treatment of subcutaneous abdominal wound healing impairment after surgery. Furthermore it will collect data that will provide a basis for subsequent efficiency analyses. Moreover, this trial seeks to evaluate the use of V.A.C.® Therapy in the acute to post-acute environment, particularly because wound healing occurs across the continuum of care and often continues after a patient is released from the hospital to homecare or other community-based care. Thus, there is a need to identify the outcomes and benefits of V.A.C.® Therapy when compared to SCWT when used across these care settings.

This trial is designed to comply with all quality requirements of IQWiG and G-BA as well as other European authorities.

Study results aspire to contribute to the benefit assessment of the IQWiG and the final decision of the G-BA regarding the eligibility of V.A.C.® Therapy to be a standard treatment recognized by German health insurance companies for full reimbursement in both medical sectors.

## Methods/design

The SAWHI-V.A.C.®-study is designed as a multinational, multicenter, randomized controlled, clinical superiority trial, with blinded photographic analysis of the primary endpoint. The trial will be performed in Germany, the Netherlands, Belgium, United Kingdom and Austria.

### Phase and classification of the trial

The study is classified as an examination of the clinical application of a CE-marked medical device with risk category IIb.

According to the German Medical Device Act (MPG) the trial is classified as an exemption to clinical investigation according to section (§) 23b MPG. This is due to the fact, that the investigational device is CE-marked, will only be operated within intended use and there will be no additional invasive or otherwise straining examinations. Examination results will only be documented if collected within clinical routine.

Ethical approval of the main ethical committee (EC): Ethical Committee of the University of Witten-Herdecke, has been fully granted without any conditions. Due to performing the trial according to section (§) 23b MPG, further participating centers in Germany will only receive a consultation for the respective main clinical investigator according to professional law. Ethical approval of participating centers in Germany is not applicable. Furthermore, until the actual time point, full EC approval was granted by the EC of the Academisch Ziekenhuis Maastricht (Netherlands) and by the NRES Committee East Midlands - Leicester, England (NHS). From each trial participant, a written informed consent will be obtained under the conditions set forth in ICH-GCP guidelines.

### Randomization

Randomization to treatment arms will be performed at a 1:1 ratio. Randomization will be performed via a centralized system with an Internet-based tool. Patients will be randomized dynamically. The permuted block sizes will randomly vary between 2, 4, and 6.

### Stratification

Stratification will take place by study center and then also subsequently by wound size (cm^3^) within study center. Wound size categories or strata to be considered will be wounds ≤ 60 cm^3^ and wounds > 60 cm^3^.

### Primary endpoint

The primary endpoint of this trial is defined as time (number of days) to achieve complete wound closure in study participants where closure was observed on or before day 42 and was confirmed to have been sustained for a minimum of 14 subsequent days. Complete wound closure is defined as 100% epithelialization, no drainage from the wound, no need for adjuvant therapy or dressing and no presence of sutures. Complete wound closure has to be confirmed after a minimum of 14 consecutive days. The clinically determined primary endpoint will be verified through blinded assessment of wound photos by observers that are independent from the clinical trial.

### Secondary clinical endpoints

The incidence of wound closure achieved within each treatment arm will be evaluated after a maximum study observation/treatment period of 42 days. These wound closures have to be confirmed as being sustained for a minimum of 14 subsequent days. This will be conducted via blinded photographic analysis. Recurrence of wound opening after initial closure and confirmed wound closure will be assessed and compared between the treatment groups. Reduction of wound size over time will be evaluated using reduction in wound volume and wound surface area over time.

### Safety endpoints

Incidence of serious adverse events (SAEs) including mortality of any cause within 132 days from the time of initiation of therapy, incidence of device-related events (ADEs), wound-related adverse events (AEs) and incidence of unsustained closure within the study treatment time of 42 days are considered to be safety endpoints of this trial.

### Patient reported outcome

Instruments and assessments used for measurement of Patient Reported Outcome (PRO) of the SAWHI-study focus on the constructs ‘Quality of life’ (QoL) and ‘Reports and Ratings of health care’ (Patient Satisfaction). QoL is measured by using a multidimensional questionnaire assessing a combination of aspects of impairments and disability and reflects a patient’s health status. QoL will be measured using the SF-36v2 questionnaire at Day 42 (end of maximum treatment/observation time) or at wound closure visit, hospital discharge, and at the general follow-up visit. Patient Satisfaction covers a structured participant self-report and a valuation of the disease-specific healthcare [29]. Furthermore, this point includes participants’ valuation of the treatment result with regard to scarring and the cosmetic result. The participant will be asked about the general estimation of therapy progression and detailed treatment satisfaction. This item will be evaluated by using a self-constructed questionnaire on the basis of specific scales of the Cologne Patient Questionnaire [30]. The Cologne Patient Questionnaire has formerly been used to develop a theory-based and empirically tested instrument for measuring patient-reported ‘psychosocial care by physicians’ [31] and represents an ideal basis for development of a specific questionnaire for participant satisfaction of this trial. Patient Satisfaction will be evaluated during general follow-up visits. Furthermore, during study visits, participants will be asked to provide their assessments of pain with a numerical rating scale. Participants will be asked to provide an estimate of their wound-associated pain of the last 24 hours.

### Health economic endpoints

Additionally to the evaluation of clinical treatment effectiveness, a prospective assessment of health economic issues will be performed. This evaluation includes the assessment of parameters relevant for inpatient and outpatient resource use (resource utilization). A detailed baseline data acquisition regarding underlying disease and intervention will create the basis for further assessments of health development and resource use. Participants’ co-morbidities will be documented using a separate, standardized case report form (CRF) sheet. The category direct medical resource use (direct medical costs) includes the resource use that results directly from wound treatment or wound-specific therapy. General and specific clinical diagnostics, relevant medication (in particular analgesics and antibiotics), specific wound therapy, number and type of debridement, residential treatment and medical attendance, outpatient treatment and medical attendance will be analyzed. Surgical reinterventions and rehospitalizations are especially a burden for health care and will be evaluated during the study. Resource use (cost) that is not directly associated with disease treatment is considered as indirect resource use (cost). This includes the evaluation of restrictions of productivity besides absence from the workplace (activities of daily living) and nonproductive time for those who are gainfully employed.

### Blinding

Due to the physical differences between the treatment regimens, it is not possible to blind either participant or physician to the treatment. To address issues of blinding, wound photo documentation will be obtained during the trial and confirmation of wound closure will be assessed via blinded assessment of photos by independent observers, which will serve as the method of primary endpoint analysis.

### Setting

**Figure 1:**
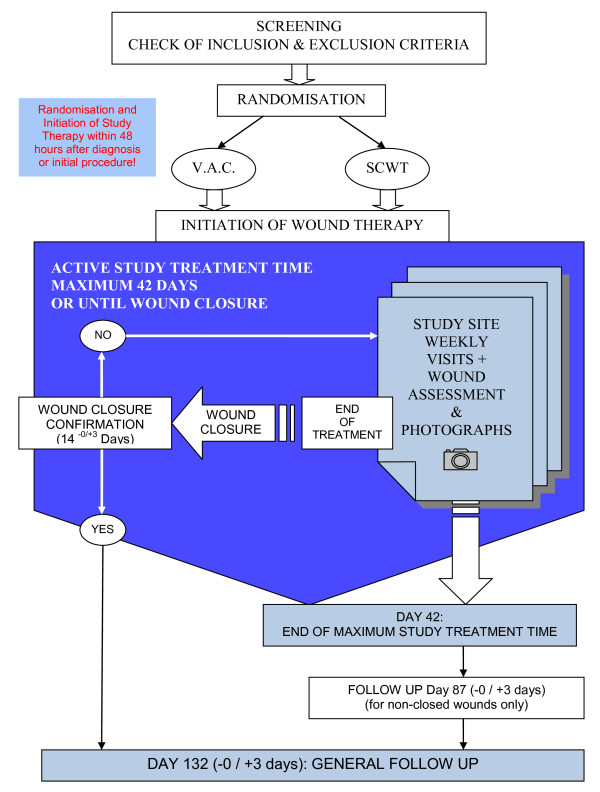
**SAWHI-study flow chart.** The figure shows the SAWHI overall study flow.

This multicenter study will be conducted in abdominal surgical hospital departments with the required manpower as well as structural and scientific qualifications. For both treatment arms, a maximum treatment time of 42 days to achieve complete wound closure shall be provided to cover 80% of participants, whether healing occurs by secondary or delayed primary closure [4]. All participants will be followed up for 90 days (three months) after the maximum treatment time, independent of whether a wound closure was achieved or not, or at which time point within the maximum timeframe for active treatment the wound closure was achieved. Thus, a general follow-up date for all study participants will be at Day 132. A follow-up period of three months after the active treatment period allows for an assessment of recurrence of the wound dehiscence or impairment as well as an adequate assessment of health economic relevant issues. Participants who have not achieved wound closure after 42 days will be seen at 45 days (six weeks) after the end of active treatment time (Day 87). This timeframe allows for a complete assessment of wound progression after study treatment and an assessment of wound closure that is achieved later than Day 42. Figure 1 gives an overview of the study flow. Study therapy will be started on participants in ambulatory care or in hospital and may be continued in ambulatory care. Due to the scope of the trial to evaluate outcomes and benefits of V.A.C.® Therapy when compared to SCWT when used across these care settings, all patients at the time point of randomization or inclusion, or during the active treatment period of 42 days that are eligible for outpatient care and have reasonable access to it, have to be transferred to outpatient care. The transfer of appropriate patients to outpatient care will be monitored and a missing transition that is not due to the medical condition of the patient or the need to provide the optimal treatment will be considered to be a protocol violation.

### Type of participants

#### ***Diagnosis***

There are two categories of wound patients with distinct post-surgical open abdominal wound diagnoses that will qualify for this study. Patients with primarily closed post-surgical abdominal wounds without fascial dehiscence that develop a spontaneous wound dehiscence or require an active reopening of the wound by the treating physician may be included in the trial. Furthermore, patients with open post-surgical abdominal wounds without fascial dehiscence that cannot be closed by primary intention and require further treatment to achieve permanent closure are appropriate for trial participation.

### Inclusion and exclusion criteria

Only patients meeting all inclusion criteria and no exclusion criteria should be included in the study. No deviation will be granted for waiver of inclusion criteria and/or exclusion criteria. Table 1 gives an overview of the inclusion and exclusion criteria of the trial. Patients to be included in the SAWHI-study must have a post-surgical abdominal wound with intact fascia and be willing and able to provide written informed consent. At time of inclusion the minimum wound size must be eligible for both possible study treatment arms according to the therapeutic requirements of the respective randomized treatment arm. Inclusion, randomization, adequate wound pre-treatment (debridement) and start of therapy must be performed within 48 hours after reopening of the wound, diagnosis of non-closable wound or in case of spontaneous wound dehiscence.

**Table 1:** SAWHI inclusion and exclusion criteria

**Inclusion criteria**	**Exclusion criteria**
Written informed consent	Age <18 years
Diagnosis of post-surgical subcutaneous abdominal wound healing impairment	Expected non-compliance with study procedures, visit schedule, and follow-up
Minimum wound size eligible for both possible study treatment arms	Pregnancy
Inclusion, randomization and start of therapy within 48 hours after initial diagnosis	Present or nonclosable defect of the abdominal fascia
	Any pre-existing or ongoing organ system failure, that cannot be stabilized or solved by appropriate medical treatment
	Necrotic tissue with eschar present
	Non-enteric and unexplored fistulas
	Malignancy of the wound
	Use of any other device based on the principle of negative pressure wound therapy on the study wound within ≤ 8 days prior to screening
	Competing therapy and procedures at the time of inclusion

The table shows the inclusion and exclusion criteria for SAWHI-study participants.

### Exclusion criteria

A patient unable or unwilling to comply with the protocol and study-related requirements, to sign the informed consent form, or to have their legally authorized representative act as a surrogate will be excluded from study participation. If a potential study patient, in the estimation of the clinical investigator, is unable to comply, the patient must be excluded from participation.

If the defect of the abdominal fascia is present at the time point of initial diagnosis or reopening of the wound, but can be closed before randomization but within the timeframe of 48 hours, participants may be included in the study. Any concomitant therapies or procedures deviating from the clinical standard wound treatment or with investigational character (for example, use of hyperbaric oxygen therapy) are not allowed ≤ 30 days prior to screening. The need for concomitant therapies or procedures that, in the opinion of the treating physician, would directly affect wound healing, or the ability to observe wound healing, is an exclusion criterion for trial participation. Concurrent participation in another trial that directly interferes with, or is likely to interfere in the foreseeable future with study procedures, patient’s compliance, wound healing or targeted endpoints is not allowed for participants of this trial.

### Intervention and comparison

**Figure 2:**
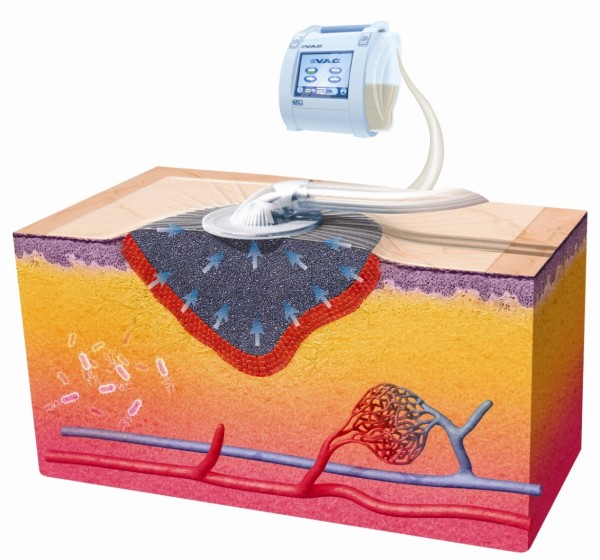
**Vacuum Assisted Closure (V.A.C.®).** The figure shows the foam dressing fitted into the wound that helps to provide the necessary mechanisms to promote granulation tissue formation. The Sensa T.R.A.C.™ pad regulates pressure at the wound site and the V.A.C.® Therapy Unit provides intermittent and continuous therapy with integrated patient safety features. Copyright by KCI with permission for use for study related publications.

This study is designed to evaluate treatment effects of a medical device in comparison to control therapy. Trial intervention is wound treatment with V.A.C.® using the basic principle of NPWT (Figure 2). The V.A.C.® therapy system is a non-invasive wound therapy system that uses controlled, localized negative (that is, subatmospheric) pressure to create an environment that promotes wound healing in chronic and acute wounds. The V.A.C.® therapy system label has been on the market since 1995 and bears the CE mark and includes the trade name and address of the manufacturer, the batch code, and all other essential information according to European Union (EU) Directive 1993/42/EEC. The V.A.C.® therapy system promotes wound healing through optimization of blood flow, decreasing local tissue edema, and removing excessive fluid from the wound bed. These physiologic changes facilitate the removal of bacteria from the wound. Additionally, the cyclical application of subatmospheric pressure alters the cytoskeleton of the cells in the wound bed through microstrain, triggering a cascade of intracellular signals that increases the rate of cell division and subsequent formation of granulation tissue. Moreover, this system has been shown to also cause macrostrain, bringing the edges of the wound closer and also stimulating wound bed granulation. [11] The V.A.C.® therapy devices that will be used in this study include the ActiV.A.C.® Therapy System, InfoV.A.C.® Therapy System, V.A.C. Freedom® ,V.A.C. Via® and V.A.C. Ulta® Therapy Systems. All V.A.C.® Therapy devices to be utilized in this study bear the CE mark and will be operated within normal conditions of use. In addition, all dressings to be used in the treatment group (V.A.C.® Therapy) bear the CE mark and will be used according to clinical guidelines. The use of the allocated V.A.C.® Therapy System should be performed according to the clinical guidelines released by KCI. Devices can be used with the black GranuFoam™ Dressing, V.A.C. GranuFoam Silver® Dressing, or V.A.C.® White Foam Dressing as indicated by the treating physician. Dressing changes are recommended to be performed every 48 to 72 hours, no fewer than three times per week, with frequency adjusted by the clinician as appropriate. Furthermore any concomitant therapies or procedures deviating from the clinical standard wound treatment or with investigational character (for example, use of hyperbaric oxygen therapy) are not allowed during the course of the trial.

Incidents, complications or adverse reactions like pain during dressing changes, maceration, cellulitis or bleeding, and so on, have been reported only sporadically while using V.A.C.® therapy for treating acute and chronic wounds. Potential adverse events can usually be avoided or significantly reduced by complying with the basic rules of vacuum therapy (as detailed in the manufacturer’s instructions for use and guidelines for the use of V.A.C.® Therapy) as well as by the use of specifically designed vacuum device systems [32-34].

Control therapy is defined as any standard conventional wound therapy (SCWT) according to local clinical standards. Standard therapy is defined as the currently accepted and widely used treatment for the respective wound type, based on the results of past research. Therapy options for standard wound care are treatments that experts agree to be appropriate, accepted, and widely used. Health care providers are obligated to provide patients with best practice and standard of care.

### Type of analysis

The primary efficacy parameter, time to complete wound closure, will be compared between the two treatment groups using a log-rank test. In addition, as a supportive analysis, a Cox proportional hazards regression will also be used in order to assess if there is a difference between treatment groups while accounting for the effect of study center and wound size (appropriate baseline and medical history parameters will also be considered). The secondary efficacy parameter, incidence of complete wound closure, will be analyzed using a chi-squared test comparing the two treatment groups. As a supportive statistical analysis, logistic regression will also be implemented. The logistic regression will include treatment, study center, and wound size. Reduction in wound area (cm^2^) and wound volume (cm^3^) will be assessed using a three-factor (treatment group, study center, and time) analysis of covariance with baseline wound area (or volume) recorded at baseline to be used as a covariate. One planned interim analysis will be performed when 250 study participants have completed the first phase of the study. The interim analysis will validate the assumptions made for the study sample size calculation and will determine if sample size re-estimation is warranted.

The results of the SAWHI-V.A.C.®-study will be analyzed by the intention-to-treat (ITT) approach. The ITT analysis population will include all randomized participants who have a valid baseline and at least one valid post baseline wound assessment (examination). For the ITT analysis population, participants will be assigned to the treatment group based on the randomization schedule, regardless of the treatment actually received. For the analysis of the primary efficacy endpoint, missing values will be incorporated as censored values. For secondary endpoints, methods for imputing missing or partially missing data will be used as appropriate. The last observation carried forward (LOCF) method will be used for the analysis of the endpoints ‘incidence of wound closure’; ‘recurrence’ and ‘reduction of wound size’ in case of study determination or death.

As a secondary approach, a per protocol (PP) analysis will be performed without patients with any protocol deviations. Violations of inclusion and exclusion criteria; changes of randomized therapy; the use of concomitant therapies or procedures deviating from the clinical standard wound treatment or with investigational character (for example, use of hyperbaric oxygen therapy) and dressing changes not performed as recommended to be performed every 48 to 72 hours (no fewer than three times per week) will be considered as protocol deviations. Furthermore, during analysis, concomitant therapies or procedures will be evaluated regarding its impact to directly affect wound healing or the ability to observe wound healing.

### Determination of sample size

The primary efficacy endpoint is the time to achieve confirmed wound closure within 42 days of surgery (that is, wound confirmed closed after 14 consecutive days). Time to confirmed wound closure will be presented with Kaplan-Meier curves and differences between the two treatment groups will be evaluated with the log-rank test. Sample size estimation has been performed using the expected difference in wound closure rates rather than hazard ratios. Assuming a complete wound closure rate of 50% in the control group, and a minimum difference of 12.5% after a treatment time of 42 days, a number of 246 participants per group would be needed (chi-squared test) to achieve 80% power with α = 0.05. If α = 0.048 due to one interim analysis (see below) sample size per group would increase only marginally (n = 249 per group, or n = 498 in total). Assuming a maximum loss-to-follow-up rate of 10% for both treatment groups, a total of 550 participants will be required. Up to 600 participants will be enrolled in this study. The computer program of Dupont and Plummer was used for sample size calculation [29]. One interim analysis will be performed when 250 participants complete the first phase of the study, and type I error rate will be adjusted using the O’Brien-Fleming method (α = 0.005 for the interim analysis and α = 0.048 for the final analysis) [35].

The final analysis is planned to be performed with time-to-event data (survival curves; log-rank test) rather than wound closure rates. However, since this more detailed approach is able to detect differences more sensitive than event rates, the calculated sample size is considered to be sufficiently large.

## Discussion

### Justification for target study population

Wounds with healing impairment after abdominal surgery and intact fascia were chosen as the target study population because they are considered to be a fair representation of open wound types and also are frequently associated with the types of comorbidities observed with other types of open wounds.

### Wound closure

Wound closure can be achieved by delayed primary intention (secondary suture, skin flap, skin graft) or secondary intention according to requirements of the participant and wound in the estimation of the attending physician. Optimal care for the participant has to be ensured.

Independent from type of closure, criteria for wound closure have to be reached within a maximum time frame of 42 days and wound closure has to be confirmed for a minimum of 14 consecutive days (14 days -0 / + 3).

### SAWHI-website, photo documentation platform and verification of clinical wound size estimation

A study-website has been set up that contains all relevant information about the trial as well as a personal login area for each participating study center and a web-based photo documentation platform for wound photographs of study participants. The website can be found using the uniform resource locator http://www.sawhi.com. Wound photographs will be uploaded by study sites for each trial visit. The Wound Healing Analyzing Tool (WHAT) has been integrated within the platform. A centralized wound analysis of anonymized photographs (blinded assessment of wound photos) will be performed, which facilitates the objective evaluation of wound healing processes. Secondary endpoints (numerical parameters) like circumference (mm), area (mm^2^), maximum length (mm), and height (mm) will be calculated from the wound image using WHAT whereas the application of the system also will be performed by blinded independent assessors.

### Data Quality Assurance

In order to guarantee the high quality of the study data and data retrieval, all participating centers will be visited on a regular basis onsite by the clinical research associates (CRAs) according to a predefined monitoring plan. Data protection rights will be respected. Participant files will be monitored on a 100% basis to control original data and to verify accurate data registration and management (100% source data verification).

### Ethical conduct of the study

This study is to be conducted in accordance with the International Conference on Harmonization (ICH) Harmonized Tripartite Guidelines for Good Clinical Practice 1996, the European Union (EU) Directive 95/46/EC on the protection of individuals with regard to the processing of personal data and on the free movement of such data as transposed into national law, the EU Medical Device Directive 93/42/EC as amended by Directive 2007/47/EC as amended into national law, the International Organization for Standardization (ISO) 14155 related to AE definitions and in the spirit of the Declaration of Helsinki concerning medical research in humans (latest edition).

### Publication

Both positive and negative results of the trial will be disclosed. The results of the trial will be submitted for publication to peer-reviewed scientific journals and/or presented at international scientific congresses. The Principal Coordinating Investigator and the Steering Committee are responsible for the primary and secondary publications and/or presentations arising from the study. KCI guarantees IFOM the publication rights for study results. KCI is entitled to examine the manuscript prior to publication and to make comments on it. KCI may delay publication for up to three months after analyzing the research results if it is applying for a patent or other important reasons. All publications will maintain data protection of participant data as well as data of the participating investigators. The publishing of data from a single study center is permitted after analysis and primary publication of the final results only. Publication of study results or data, including data of a single study center, has to be reviewed and approved by the Steering Committee, IFOM, and KCI.

### Trial status

Recruiting.

## Abbreviations

AE: Adverse events; CRF: Case report form; EU: European Union; G-BA: German: Gemeinsamer Bundesausschuss; : English: Federal joint committee; ICH: International conference of harmonization; IFOM: Institut für Forschung in der Operativen Medizin; IQWIG: German: Institut für Qualität und Wirtschaftlichkeit im Gesundheitswesen; : English: Institute for quality and efficiency in health care; ISO: International Organization for Standardization; ITT: Intention-to-treat; KCI: Kinetic concepts incorporated; LOCF: Last observation carried forward; MPG: German: Medizinproduktegesetz; : English: German medical device act; NPWT: Negative pressure wound therapy; PP: Per protocol; PRO: Patient reported outcome; QoL: Quality of life; RCT: Randomized controlled trial; SAE: Serious adverse events; SCWT: Standard conventional wound therapy; V.A.C.®: Vacuum Assisted Closure (registered trade mark); WHAT: Wound healing analyzing tool.

## Competing interests

The authors declare that they have no personal competing interests.

## Authors’ contributions

DS contributed to the study by conception and design. She drafted the manuscript of the article. RL contributed to the development of the protocol, did set up the statistical analysis and provided critical revision of the manuscript. EN contributed to the development of the protocol. He provided critical revision of the manuscript. All authors read and approved the final version of the manuscript.
